# Two adult cases of canal of Nuck cyst: diagnosis and treatment

**DOI:** 10.1093/jscr/rjaf217

**Published:** 2025-04-23

**Authors:** Moath Hattab, Muhammad Takhman, Laith M Daraghmeh, Qutaiba Mahmoud, Hanood Abu-Ras, Alaa Rostom

**Affiliations:** Faculty of Medicine and Health Sciences, Department of Medicine, An-Najah National University, Old Campus Street, Nablus P400, Palestine; Faculty of Medicine and Health Sciences, Department of Medicine, An-Najah National University, Old Campus Street, Nablus P400, Palestine; Department of General Surgery, An-Najah National University Hospital, Asira Street, Nablus P400, Palestine; Department of Radiology, An-Najah National University Hospital, Asira Street, Nablus P400, Palestine; Department of Pathology, An-Najah National University Hospital, Asira Street, Nablus P400, Palestine; Faculty of Medicine and Health Sciences, Department of Medicine, An-Najah National University, Old Campus Street, Nablus P400, Palestine; Department of General Surgery, An-Najah National University Hospital, Asira Street, Nablus P400, Palestine

**Keywords:** canal of Nuck, hydrocele, hernia, inguinal swelling, case report

## Abstract

Abnormalities of the canal of Nuck are rare congenital defects in females, typically repaired in childhood. Canal of Nuck cysts or hydroceles often present as fluctuating inguinal masses, commonly misdiagnosed as hernias. Ultrasonography is key for accurate diagnosis, and surgical excision is usually the primary treatment. We present two cases of canal of Nuck cysts in female patients, aged 28 and 32, with no significant past medical history. The 28-year-old underwent surgical excision, while the 32-year-old opted for a watchful waiting approach. Both cases had unremarkable follow-up, with no complications. This report highlights the importance of considering a broad differential diagnosis when evaluating inguinal masses or pain, including canal of Nuck.

## Introduction

Hydrocele of the canal of Nuck is a rare condition caused by an embryogenic defect in females, where the canal of Nuck (the female counterpart of the male’s processus vaginalis) fails to fully close. While the canal typically obliterates during the first year of life, in some cases, only the proximal part closes, leaving the distal part patent. This results in the peritoneum extending into the inguinal canal, leading to the accumulation of serous fluid and the formation of a hydrocele of the canal of Nuck [1–3].

This defect often remains undetected unless it causes complications such as hydrocele or varicocele. A hydrocele results in a fluctuating inguinal mass, which may be painful or painless and is often mistaken for a hernia or cyst. Careful physical examination can suggest alternative diagnoses, but imaging is necessary for confirmation. Hydroceles do not enlarge with a cough impulse, unlike hernias, and extend toward the labia majora. They also do not produce bowel sounds on auscultation unless complicated by a hernia. Other differential diagnoses include leiomyomas, sarcomas, cysts, abscesses, and lymphadenitis, each with distinct clinical features.

Two cases of hydrocele of the canal of Nuck were diagnosed through clinical history, physical examination, and ultrasound. One case was treated surgically, while the other opted for non-surgical management with watchful observation. This report aims to contribute to the literature on this rare condition. The cases were managed at a non-profit medical and academic institution and follow the SCARE Criteria [4].

## Presentation of case

### Case 1

**Figure 1:**
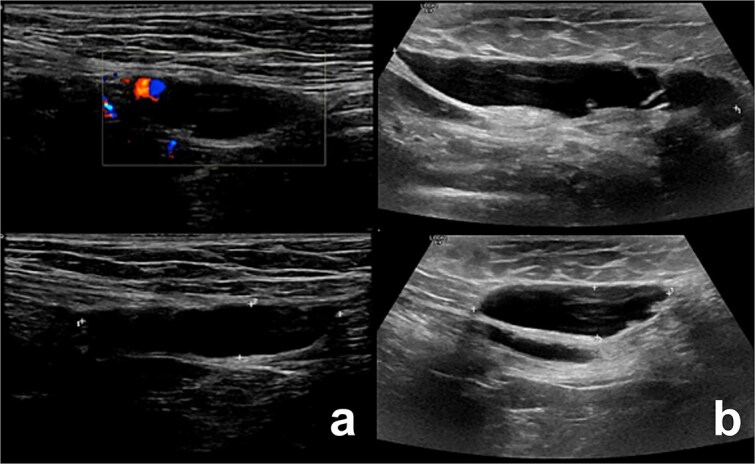
Illustrates the ultrasound findings for the two patients, highlighting the presence of the canal of Nuck cyst (case 1: a, case 2: b).

A 28-year-old woman presented with a 6-week history of a progressively enlarging, painful right groin lump. Examination revealed a small, mobile lump in the right inguinal region without erythema or tenderness. Ultrasound showed a 3 × 1.8 cm septated cystic lesion in the inguinal canal, with no hernia. Aspiration reduced its size to 1.8 × 0.9 cm with slight symptom relief, but the lesion recurred, enlarging to 3.5 × 1.0 cm after 2 weeks, causing radiating pain to the medial thigh. Follow-up ultrasound showed recurrence (Fig. 1a), prompting surgical exploration.

**Figure 2:**
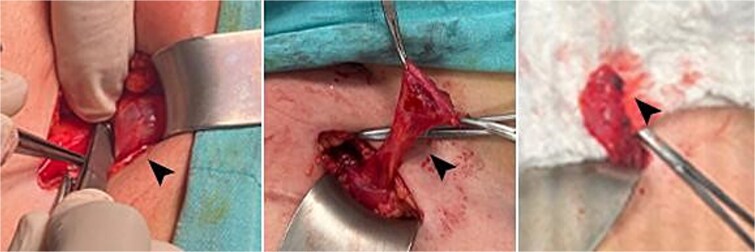
Intraoperative images showing the cyst, highlighted by the head arrow.

**Figure 3:**
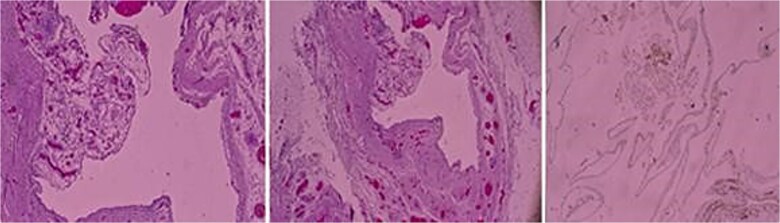
Three histopathologic microscopic images of the cyst.

A right inguinal exploration was performed under general anesthesia. A cystic structure consistent with a canal of Nuck cyst was identified within the inguinal canal (Fig. 2). Dissection was performed around the cyst until it was completely released. The cyst was excised in its entirety and sent for histopathological analysis that confirmed the diagnosis of a benign cyst lined with mesothelial cells and containing serous fluid, consistent with a canal of Nuck cyst (Fig. 3). Postoperatively, the patient recovered well, tolerated her diet, ambulated independently, and maintained clean dressings.

### Case 2

A 32-year-old female with no significant past medical history presented with a 2-week history of inguinal pain, primarily during walking, occasionally radiating to the medial thigh. Clinical examination revealed point tenderness over the mid-inguinal ligament, with no palpable swelling and a negative cough impulse.

Ultrasound showed a 2.5 × 1.1 × 3.5 cm cystic lesion, without soft tissue components, located superficially and medial to the pubic bone at the level of the superficial inguinal ring. There was no communication with the peritoneum. These findings, as shown in Fig. 1b, were suggestive of a canal of Nuck cyst.

The patient declined surgical excision and chose watchful observation. Over a 2-month follow-up period, she reported no new complaints or symptom progression.

## Discussion

The canal of Nuck, an embryological anomaly in females, occurs when the processus vaginalis fails to close, creating a communication between the peritoneal cavity, inguinal canal, and labia majora. While rare, it is more commonly seen in children and is increasingly diagnosed in adults due to improved imaging. If patent, it can lead to inguinal hernias. Differential diagnoses include lymph nodes, cysts, hernias, infections, endometriosis, and tumors [5].

There are three types of hydrocele in the canal of Nuck: the common type, with no communication to the peritoneal cavity; the second type, with persistent communication; and the hourglass type, a combination of both, where the hydrocele is partly enclosed and partly communicating due to inguinal ring constriction [6].

Various terms, such as “canal of Nuck cyst,” “Nuck cyst,” and “hydrocele of the canal of Nuck,” are used interchangeably to describe this condition. Due to its rarity, there is no consensus on the best diagnostic or treatment approach, and most available data come from case reports and series.

The canal of Nuck cyst commonly presents as an inguinal or genital swelling, which can be painful or painless. In some cases, the mass may be reducible manually and remains unchanged during the Valsalva maneuvre, though it may enlarge when standing. Differentiating it from other swellings based on clinical symptoms alone is difficult. Ultrasound is the initial imaging method, showing a thin-walled, anechoic or hypoechoic structure without vascular flow or changes during the Valsalva maneuvre. MRI is the preferred method for detailed evaluation, showing a thin-walled hypointense mass on T1-weighted and hyperintense mass on T2-weighted sequences, with no contrast enhancement. CT is rarely used due to radiation concerns, especially in children, but can show a fluid-filled sac without enhancement. MRI is preferred for its better detail and safety [7].

Due to the rarity of canal of Nuck cysts, no standard treatment exists. Conservative options like aspiration or sclerotherapy are reported, but hydrocelectomy is generally recommended, with or without ligation of the cyst [7].

Management involves either open or laparoscopic excision of the cyst, with possible primary closure of the inguinal defect, often using mesh. The choice between these approaches depends on factors such as the accuracy of the preoperative diagnosis, the presence of an inguinal hernia, and the extent of the disease. Inguinal hernia repair, with or without mesh, can also be safely performed during the procedure [8].

Of the two cases, one was successfully treated with surgical excision, while the other opted for watchful waiting, avoiding surgery or aspiration, and requiring follow-up to monitor progression or resolution.

## Conclusion

The canal of Nuck cyst is a rare condition, often overlooked in older females. More research and case studies are needed to improve diagnosis and treatment. Clinicians should stay vigilant when assessing inguinal masses for better outcomes.

## Highlights

- Canal of Nuck cysts are rare congenital anomalies in females caused by incomplete obliteration of the processus vaginalis.
- Surgical excision is considered the definitive treatment for a canal of Nuck cyst.
